# Influential factors of the prognosis of patients with winter sports-induced traumatic cervical spinal cord injury complicated with intramedullary hemorrhage and edema after emergency surgical treatment

**DOI:** 10.12669/pjms.40.4.7644

**Published:** 2024

**Authors:** Peinan Zhang, Xinming Yang, Yanlin Yin, Yao Yao

**Affiliations:** 1 Peinan Zhang, Department of Orthopedic, The First Hospital Affiliated to Hebei North University, Zhangjiakou 075000, Hebei, China; 2 Xinming Yang, Department of Orthopedic, The First Hospital Affiliated to Hebei North University, Zhangjiakou 075000, Hebei, China; 3 Yanlin Yin, Department of Orthopedic, The First Hospital Affiliated to Hebei North University, Zhangjiakou 075000, Hebei, China; 4 Yao Yao, Department of Orthopedic, The First Hospital Affiliated to Hebei North University, Zhangjiakou 075000, Hebei, China

**Keywords:** Winter sports, Traumatic cervical spinal cord injury, Intramedullary hemorrhage, edema, Emergency surgery, Prognosis

## Abstract

**Objective:**

To explore relevant influencing factors of the prognosis of patients with winter sports-induced traumatic cervical spinal cord injury complicated with intramedullary hemorrhage and edema after emergency surgical treatment.

**Methods:**

A retrospective analysis was performed on 73 cases of traumatic cervical spinal cord injury complicated with intramedullary hemorrhage and edema in The First Hospital Affiliated to Hebei North University from January 2020 to October 2022. The enrolled patients were divided into the good prognosis (n=17) group and poor prognosis (n=56) group according to the recovery of neurological function after six months of follow-up. The risk factors affecting the prognosis of patients with traumatic cervical spinal cord injury complicated with intramedullary hemorrhage and edema after emergency surgery were analyzed by binary Logistic regression.

**Results:**

Among the enrolled 73 patients with traumatic cervical spinal cord injury complicated with intramedullary hemorrhage and edema, 56 cases showed significant improvement in ASIA Grade-6 months after operation, with an improvement rate of 76.71%. Further Logistic regression analysis revealed that concomitant diabetes, preoperative MSCC>40.83% and recovery rate of AMS <40.13% 3d after operation were independent risk factors affecting the poor prognosis of patients with traumatic cervical spinal cord injury complicated with intramedullary hemorrhage and edema.

**Conclusions:**

Emergency surgery can improve the neurological function of patients with cervical spinal cord injury complicated with intramedullary hemorrhage and edema caused by winter sports. Concomitant diabetes, preoperative MSCC and recovery rate of AMS 3d after operation are the main factors affecting the prognosis of patients with emergency surgery.

## INTRODUCTION

The popularity of winter sports has burgeoned with the opening of the world-famous Beijing Winter Olympic Games. There is a dramatic increase in the number of winter sports enthusiasts in the outdoor ice-snow tourism venues and winter sports venues in various regions, accompanied by significant increase in related sports injuries.^1^ According to a large number of reports, the head, neck and knee joints are the most vulnerable parts in winter sports, the most serious of which is traumatic spinal cord injury.^2^ Traumatic spinal cord injury, occurring possibly in the cervical, thoracic, lumbar other spinal cord segments, refers to a damage to the structure and function of the spinal cord caused by direct or indirect external forces.^3^ Cervical spinal cord injury is the most severe traumatic spinal cord injury, and common causes of injury include traffic accidents, falls, injuries, and sports injuries caused by competitive sports such as ice and snow sports. In severe cases, it can lead to paralysis or even death of the patient’s limbs.^4^,^5^ Patients with traumatic cervical spinal cord injury may often suffer from secondary injuries such as hemorrhage and edema in the cervical spinal cord due to fracture compression. Such injuries are generally manifested as focal hemorrhage in the center of the injury, microvascular rupture in the central gray matter and extensive edema formation, which can aggravate the degree of injury to the patient’s limb motor function.^6^ Therefore, timely and effective treatment may be beneficial to improve clinical efficacy and prognosis of patients with traumatic spinal cord injury complicated with intramedullary hemorrhage and edema. In the clinical practice currently, early emergency surgery is the major therapeutic approach for traumatic cervical spinal cord injury complicated with intramedullary hemorrhage and edema, which has achieved good results.^7^

However, there are still some patients with limb movement disorder or vesicorectal disturbance after surgery, which may affect their prognosis. Previous studies have confirmed that the degree of cervical spinal cord injury, neurological function grading, concomitant injuries, underlying medical diseases, and respiratory complications during trauma are factors that affect the prognosis of emergency surgery.^8^ Accordingly, the present study was performed primarily to discuss and analyze the relevant factors that affect the prognosis for patients with winter sports-induced traumatic cervical spinal cord injury complicated with intramedullary hemorrhage and edema after emergency surgery, so as to provide reference for the prevention and treatment of traumatic cervical spinal cord injury caused by winter sports and the evaluation of the prognosis of patients receiving emergency surgery.

## METHODS

A retrospective analysis was performed on the clinical data of 73 cases of traumatic cervical spinal cord injury complicated with intramedullary hemorrhage and edema in The First Hospital Affiliated to Hebei North University from January 2020 to October 2022.

### Ethical Approval

The study was approved by the Institutional Ethics Committee of The First Hospital Affiliated to Hebei North University (No.:2022-035; Date: May 15, 2022), and written informed consent was obtained from all participants.

### Inclusion criteria

- Patients aged >18 years;
- With clear history of trauma caused by winter sports, meeting the diagnostic criteria for cervical spinal cord injury^9^ and confirmed by imaging examination; with intramedullary hemorrhage and edema shown by MRI;
- With Grade-A~C of American Spinal Injuries Association (ASIA) Impairment Scale (AIS),^10^ who successfully completed emergency surgery in our hospital;
- With complete clinical data.

### Exclusion criteria:

- Patients with severe heart, liver, kidney and other organ dysfunction and other immune-related diseases, with craniocerebral injury, with coagulation dysfunction, with AISA motor score of 96~100 points at admission;
- With postoperative survival <six months;
- With malignant tumors or psychological and mental diseases.

The enrolled patients were divided into the good prognosis (n=17) group and poor prognosis (n=56) group according to the recovery of neurological function after six months follow-up. *Evaluation criteria for good prognosis:* During the follow-up period of six months, the patients survived with the preoperative and postoperative ASIA grades recovered to at least one grade.

### Collection of clinical data

General data of these patients were collected from the electronic medical record system of our hospital, including preoperative American Spinal Cord Injury Association (ASIA) neurological function classification,^11^ intramedullary edema length (IEL) by preoperative MRI, intramedullary hemorrhage length (IHL) by MRI, intramedullary spinal cord compression (MSCC) and recovery rate of American Spinal Injury Association (ASIA) motor score (AMS),^12^ complicated with soft tissue injury or not, surgical method (anterior approach, posterior approach or combined approach), AMS recovery rate before and 3d after operation, endotracheal intubation, albumin level, postoperative rehabilitation treatment or not, AIS classification six months after operation. AMS recovery rate refers to the percentage of the difference between postoperative AMS and preoperative AMS and the difference between 100 and preoperative AMS. It can indicate the changes of motor function in patients with traumatic cervical spinal cord injury complicated with intramedullary hemorrhage and edema. The endpoint cessation of treatment of this study was significant improvement of the patient’s symptoms, without the need for further surgical intervention.

### Statistical analysis

SPSS 22.0 statistical software was employed for data processing in this study. Measurement data were expressed by (*χ̅*±*s*) and compared by independent two-sample t-test between groups. Meanwhile, counting data were described by rates, and the inter-group difference was compared by using *χ*^2^ test. The risk factors affecting the prognosis of patients with traumatic cervical spinal cord injury complicated with intramedullary hemorrhage and edema after emergency surgery were analyzed by binary Logistic regression. A two-tailed *P*<0.05 was considered to have significant difference.

## RESULTS

All the 73 patients with traumatic cervical spinal cord injury complicated with intramedullary hemorrhage and edema were followed up for 6-12 months, with an average of (10.60±1.62) months. No patients were lost to follow-up, and the follow-up rate was 100%. Three of them died within six months after operation, including one case of respiratory failure, two cases of pulmonary infection, and one case of deep venous thrombosis of lower limb, with a mortality rate of 4.11%.

Among the enrolled 73 patients with traumatic cervical spinal cord injury complicated with intramedullary hemorrhage and edema, 56 cases showed significant improvement in ASIA grade six months after operation, with an improvement rate of 76.71% (Table-I).

**Table-I:** Analysis of postoperative follow-up of patients with traumatic cervical spinal cord injury complicated with intramedullary hemorrhage and edema.

ASIA grade before operation	Number of cases before operation	ASIA Grade-6 months after operation				

		A	B	C	D	E
A	16	5	8	3	-	-
B	35	-	9	20	6	-
C	22	-	-	3	15	4
Total cases	73	5	17	26	21	4

As described in Table-II to III, the poor prognosis of winter sports-induced traumatic cervical spinal cord injury complicated with intramedullary hemorrhage and edema was associated with their age, concomitant diabetes, level of cervical spinal cord injury, preoperative IEL, preoperative IHL, preoperative MSCC and recovery rate of AMS 3d after operation (*P*<0.05). While the prognosis showed no relationship with the patient’s gender, BMI, hypertension or coronary heart disease, smoking history, time from injury to admission, time from injury to emergency surgery, complicated with soft tissue injury or not, surgical method, preoperative AMS, AIS classification six months after operation, endotracheal intubation, albumin level and rehabilitation treatment (*P*>0.05).

**Table-II:** Comparison of clinical characteristics of patients with traumatic cervical spinal cord injury complicated with intramedullary hemorrhage and edema between the poor prognosis group and the good prognosis group [cases (%), *χ̅*±*S*].

Indicators		Poor prognosis group (n=17)	Good prognosis group (n=56)	χ^2^/t value	P value
Gender (cases)	Male	10 (58.82)	36 (64.29)	0.167	0.683
	Female	7 (41.18)	20 (35.71)		
Age (years)	≤50	8 (47.06)	41 (73.21)	4.043	0.044
	>50	9 (52.94)	15 (26.79)		
BMI (kg/m^2^)	≤24	12 (70.59)	39 (69.64)	0.006	0.941
	>24	5 (29.41)	17 (30.36)		
Underlying diseases (cases)	Hypertension	10 (58.82)	16 (28.57)	3.626	0.057
	Diabetes	7 (41.18)	10 (17.86)	3.970	0.046
	Coronary heart disease	4 (23.53)	14 (25.00)	0.015	0.902
Smoking history (cases)	Without	12 (70.59)	37 (66.07)	0.121	0.728
	With	5 (29.41)	19 (33.93)		
Level of cervical spinal cord injury (cases)	>C_4_	10 (58.82)	40 (71.43)	5.205	0.023
	C_5_~C_8_	7 (41.18)	16 (28.57)		
Time from injury to admission (h)		7.72±1.25	7.31±1.38	1.095	0.277
Time from injury to emergency operation (h)		16.27±4.69	15.74±4.05	0.455	0.650
Soft tissue injury (cases)	Without	6 (35.29)	34 (60.71)	3.402	0.065
	With	11 (64.17)	22 (39.29)		
Combined injury (cases)	Rib fracture	4 (23.53)	12 (21.43)	0.652	0.722
	Limb injury	7 (41.18)	29 (51.79)		
	Others	6 (35.29)	15 (26.79)		
Preoperative AISA classification (cases)	A	5 (29.41)	11 (19.64)	0.783	0.676
	B	7 (41.18)	28 (50.00)		
	C	5 (29.41)	17 (30.36)		
Surgical approach (cases)	Anterior approach	4 (23.53)	17 (30.36)	1.528	0.466
	Posterior approach	11 (64.71)	27 (48.21)		
	Combined approach	2 (11.76)	12 (21.73)		
Endotracheal intubation (cases)	Without	11 (64.17)	36 (64.29)	0.001	0.975
	With	6 (35.29)	20 (35.71)		
Albumin (g/L)	≤30	12 (70.59)	37 (66.07)	0.121	0.728
	>30	5 (29.41)	19 (33.93)		
Rehabilitation treatment (cases)	Without	4 (23.53)	12 (21.43)	0.034	0.854
	With	13 (76.47)	44 (78.57)		

Our study further assigned the variables with differences in Table-II to III that affected the poor prognosis of patients with traumatic cervical spinal cord injury complicated with intramedullary hemorrhage and edema, with the significant level set as α=0.05. The assignment of dependent and independent variables is shown in Table-IV. Logistic regression analysis revealed that concomitant diabetes (OR=2.784, *P*=0.047), preoperative MSCC>40.83% (OR=2.707, *P*=0.021) and recovery rate of AMS <40.13% 3d after operation (OR=2.751, *P*=0.031) were independent risk factors affecting the poor prognosis of patients with traumatic cervical spinal cord injury complicated with intramedullary hemorrhage and edema. (Table-IV to V)

**Table-III:** Comparison of MRI imaging parameters of patients with traumatic cervical spinal cord injury complicated with intramedullary hemorrhage and edema between the poor prognosis group and the good prognosis group [cases (%),*χ̅*±*S*].

Indicators		Poor prognosis group (n=17)	Good prognosis group (n=56)	t value	P value
Recovery rate of AMS (%)	Before operation	30.98±4.42	31.75±4.36	0.635	0.527
	3d after operation	33.71±6.08	46.54±6.94	6.858	<0.001
Preoperative IEL (mm)		39.58±5.14	33.45±4.61	4.675	<0.001
Preoperative IHL (mm)		28.23±6.58	17.16±3.94	8.565	<0.001
Preoperative MSCC (%)		44.78±7.16	37.96±6.83	3.566	<0.001

***Note:*** AMS: ASIA motor score; IEL: tramedullary edema length; IHL: tramedullary hematoma length; MSCC: tramedullary spinal cord compression.

**Table-IV:** Assignment of research variables.

Variables	Related factors	Definition and assignment	
X_1_	Age	≤50 years=0	>50 years=1
X_2_	Concomitant diabetes	Without=0	With=1
X_3_	Level of cervical spinal cord injury	C_5_~C_8_=0	>C_4_=1
X_4_	Recovery rate of AMS 3d after operation	>40.13%=0	≤40.13%=1
X_5_	Preoperative IEL	≤21.04mm=0	>21.04mm=1
X_6_	Preoperative IHL	≤22.95mm=0	>22.95mm=1
X_7_	Preoperative MSCC	≤40.83%=0	>40.83%=1

**Table-V:** Multi-factor analysis of prognosis of patients with traumatic cervical spinal cord injury complicated with intramedullary hemorrhage and edema.

General data	β	SE	Wald χ^2^	OR	95%CI	P value
Age	0.983	0.522	3.546	2.672	0.976~7.319	0.056
Concomitant diabetes	1.024	0.514	0.526	2.784	1.017~7.625	0.047
Level of cervical spinal cord injury	0.783	0.508	2.376	2.188	0.808~5.992	0.124
Recovery rate of AMS 3d after operation	0.996	0.431	0.526	2.707	1.163~6.301	0.021
Preoperative IEL	1.035	0.541	0.526	2.815	0.975~8.128	0.056
Preoperative IHL	0.977	0.536	3.322	2.656	0.929~7.596	0.069
Preoperative MSCC	1.012	0.467	0.526	2.751	1.102~ 6.871	0.031

## DISCUSSION

The general public generally lacks professional training in ice and snow sports, which is prone to injuries to the spine, hip, and knee joints.^13^ Traumatic cervical spinal cord injury is a common spinal injury in ice and snow sports injuries. Patients are prone to motor and sensory dysfunction, as well as limb paralysis. In severe cases, it can lead to breathing difficulties due to diaphragmatic paralysis, and even suffocation and death.^14^ Intramedullary hemorrhage and edema are caused by the rupture of gray matter blood vessels in the spinal cord caused by spinal cord injury, indicating severe spinal cord injury in patients and reflecting their neurological function after spinal cord injury.^15^ Its treatment mainly aims to alleviate the progression of the disease and intervene in secondary injuries through early surgical surgery combined with drug therapy and rehabilitation treatment. However, there are many factors that affect prognosis in clinical practice, which have different effects on the recovery of spinal cord function in patients after surgery.^16^,^17^ This study mainly explores the relevant factors that affect the prognosis of emergency surgical treatment in patients with traumatic cervical spinal cord injury combined with intramedullary hemorrhage and edema caused by ice and snow sports.

The traditional view is that surgery will further exacerbate spinal cord injury, so patients with spinal cord injury are prohibited from undergoing surgical treatment. With the improvement of surgical and anesthesia techniques, surgical decompression for acute cervical spinal cord injury has been widely applied, and research has confirmed that patients can achieve good clinical results after emergency surgical treatment. The results of this study showed that among the 73 patients with traumatic cervical spinal cord injury combined with intramedullary hemorrhage and edema who were included in the study, 52 patients had a good prognosis after a 6-month follow-up, accounting for 71.23%. This is basically consistent with the research results of Sharma A et al. indicating that emergency surgery can significantly improve the prognosis of patients.^18^ Traumatic cervical spinal cord injury combined with intramedullary hemorrhage and edema can initiate secondary injury on the basis of mechanical injury, leading to peripheral neuronal and axonal damage, further exacerbating the degree of injury. ASIA injury grading is a recognized standard for evaluating the degree of spinal cord injury, which can quantitatively evaluate spinal cord injury.^19^ Surgery can relieve compression caused by spinal cord edema and hematoma, achieve stability of the cervical spine, improve blood supply to the cervical spinal cord, and promote the recovery of spinal cord nerve function in patients. In this study, 56 patients with traumatic cervical spinal cord injury caused by ice and snow sports combined with intramedullary hemorrhage and edema achieved significant improvement in ASIA grading at six months after surgery, with an improvement rate of 76.71%, indicating that emergency surgery can effectively promote the recovery of their neurological and motor functions.

The neurological recovery of patients with traumatic cervical spinal cord injury may be unpredictable, and there are many factors that affect the patient’s neurological recovery. Older patients often experience osteoporosis and a significant decrease in immune defense function, which is not conducive to the recovery of spinal cord injury. In patients with diabetes, abnormal glucose metabolism leads to high glucose status in the body, which has a certain killing and damage effect on nerve cells and can affect the functional status of the spinal cord.^20^ MRI can clearly display the relationship between the spinal cord and surrounding tissues, making it the best imaging basis for evaluating neurological impairment. It is characterized by edema signals or abnormal high signals in the bone marrow, which is a typical manifestation of bone contusion and often indicates poor prognosis.^21^ Preoperative IEL, IHL, and MSCC are common MRI evaluation indicators for spinal cord injury, while AMS is the MRI score for postoperative spinal cord compression signs, The higher the score, the more severe the degree of spinal cord compression in imaging. The recovery rate of AMS is a reliable indicator of postoperative imaging improvement and prognosis evaluation in cervical spinal cord injury. The severity of nerve injury in patients with traumatic cervical spinal cord injury combined with intramedullary hemorrhage and edema is closely related to the degree of spinal cord compression. Spinal cord compression exceeding 20% can significantly reduce spinal cord conduction function.^22^,^23^ The recovery rate of AMS at three days after surgery is a predictive factor for neurological function recovery at six months after surgery in patients with traumatic cervical spinal cord injury accompanied by intramedullary hemorrhage and edema. The results of this study show that the poor prognosis of patients with traumatic cervical spinal cord injury complicated with intramedullary hemorrhage and edema is related to their age, diabetes, cervical spinal cord injury level, preoperative IEL, preoperative IHL, preoperative MSCC and postoperative recovery rate of 3 days AMS. Logistic regression analysis showed that diabetes, preoperative MSCC and postoperative recovery rate of AMS were independent risk factors affecting the prognosis of emergency surgery in patients with traumatic cervical spinal cord injury complicated with intramedullary hemorrhage and edema.

### Limitations of study

It includes small sample size, the absence of analysis of the impact of different emergency surgical methods and severity of the disease on the prognosis of patients, which require in-depth verification based on expanded sample size in the future.

## CONCLUSIONS

Emergency surgery can improve the neurological function of patients with cervical spinal cord injury complicated with intramedullary hemorrhage and edema caused by winter sports. Concomitant diabetes, preoperative MSCC and recovery rate of AMS 3d after operation are risk factors affecting the prognosis of patients with emergency surgery.

### Authors’ Contributions:

**PZ** and **XY:** Conducted the study, collection of data, drafted the manuscript, are responsible and accountable for the accuracy and integrity of the work.

**YY:** Statistical analysis and participated in its design.

**YY:** Acquisition, analysis, and interpretation of data and drafting the manuscript.

All authors read and approved the final manuscript.
